# NRF2 Antioxidant Response and Interferon‐Stimulated Genes Are Differentially Expressed in SARS‐CoV‐2‐Positive Young Subjects

**DOI:** 10.1002/iid3.70109

**Published:** 2025-01-14

**Authors:** Toscanelli Walter, Fracella Matteo, De Angelis Marta, Scagnolari Carolina, Sorrentino Leonardo, Piselli Elena, Marcocci Maria Elena, Midulla Fabio, Mancino Enrica, Nenna Raffaella, Petrarca Laura, Palamara Anna Teresa, Antonelli Guido, Pierangeli Alessandra, Nencioni Lucia

**Affiliations:** ^1^ Laboratory Affiliated to Istituto Pasteur Italia‐Fondazione Cenci Bolognetti, Department of Public Health and Infectious Diseases Sapienza University Rome Italy; ^2^ Laboratory of Virology, Department of Molecular Medicine Sapienza University Rome Italy; ^3^ Department of Maternal Infantile and Urological Sciences Sapienza University Rome Italy; ^4^ Department of Infectious Diseases Istituto Superiore di Sanità Rome Italy

**Keywords:** antioxidant response, APE‐1, inflammasome, Interferon response, NRF2, SARS‐CoV‐2

## Abstract

**Background:**

Several respiratory viruses, including Severe Acute Respiratory Syndrome‐Coronavirus‐2 (SARS‐CoV‐2), suppress nuclear factor‐E2‐related factor‐2 (NRF2) antioxidant response, generating oxidative stress conditions to its advantage. NRF2 has also been reported to regulate the innate immune response through the inhibition of the interferon (IFN) pathway. However, its modulation in younger individuals and its correlation with the IFN response remain to be elucidated.

**Methods:**

The NRF2 and redox‐related genes expression was examined in nasopharyngeal swabs from children attending the pediatric hospital for SARS‐CoV‐2 molecular testing. Expression levels were analyzed by stratifying the population according to the SARS‐CoV‐2 positivity, age, or the presence of symptoms. The results were correlated with Types I and III IFN genes and IFN‐stimulated genes (ISGs).

**Results:**

We found that NRF2 expression was markedly diminished in positive patients compared to negative. Moreover, it correlated with higher expression of IFNα2 and IFNλ3, as well as ISG15 and ISG56. Interestingly, symptomatic patients with anosmia/ageusia showed pronounced expression of apurinic/apyrimidinic endonuclease1/redox factor 1 (APE1), together with Type I IFNs, ISG56, and the inflammasome component NLRP3.

**Conclusion:**

The results indicate an interdependence between NRF2 antioxidant pathway and IFN‐mediated response during SARS‐CoV‐2 infection in young subjects.

## Graphical abstract

This study highlights the role of NRF2 and related genes in the regulation of the IFN response in SARS‐CoV‐2‐positive children. We confirmed that SARS‐CoV‐2 infection inhibits the NRF2‐mediated antioxidant response in the upper respiratory tract also in pediatric subjects, although NRF2 was less expressed in asymptomatic subjects compared to groups with mild symptoms, in which inflammatory genes were upregulated. The observed suppression of NRF2 and its correlation with high increases of IFNα2 and IFNλ3 response, along with increased expression of ISGs, provide valuable insights into the host response to the virus in the young population. Further investigations are warranted to explore the potential of NRF2 modulators as therapeutic strategies for COVID‐19, particularly, in children.

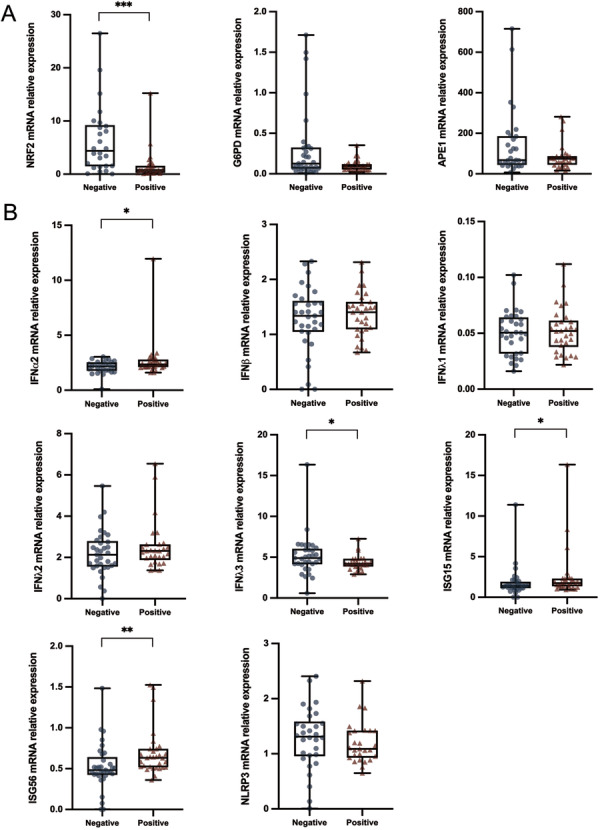

## 1 Introduction

The recent pandemic caused by SARS‐CoV‐2 has highlighted the importance of respiratory viruses spreading between humans, especially in critically ill infected patients [1]. Severe forms usually occur in the elderly or in individuals with comorbidities. In relation to children, most of them remain asymptomatic to SARS‐CoV‐2 infection [2], although the pathogenesis of this virus in young individuals remains to be investigated.

Since viruses use cell machinery to replicate their genome and produce viral proteins, several intracellular factors, including the redox state, may directly or indirectly affect the progression and outcome of viral infection [3]. In physiological conditions, the redox balance between oxidant and antioxidant species is maintained by enzymatic and nonenzymatic systems, and it finely regulates several cell functions. Redox homeostasis is maintained by intracellular glutathione (GSH) levels and by cellular enzymes such as superoxide dismutases, catalase, and GSH peroxidase. Additionally, the NRF2 transcription factor participates in antioxidant defense systems by regulating the transcription of many genes encoding antioxidant enzymes, through the antioxidant response element (ARE) binding sites in their promoter regions [4]. Under physiological conditions, NRF2 is retained in the cytosol and is controlled by Kelch‐like ECH‐associated protein 1 (KEAP1), which targets NRF2 for ubiquitination and subsequent degradation by proteasomes. In the presence of augmented reactive oxygen species (ROS) production, ROS interact with KEAP1, preventing NRF2 degradation, which, in turn, translocates to the nucleus and binds to the conserved ARE region to activate a battery of antioxidative and cellular defense targets [5, 6]. It is known that different viruses break redox equilibrium and induce oxidative stress, which, in turn, facilitates specific steps of the virus life cycle and activates an inflammatory response [3]. As an example, the influenza virus induces oxidative stress by increasing ROS production, depleting the intracellular GSH content, and activating specific redox‐sensitive enzymes to ensure its replication into the host cell [7, 8, 9, 10]. Additionally, we recently demonstrated that NRF2 and its related gene glucose‐6‐phosphate dehydrogenase (G6PD) are downregulated at both mRNA and protein levels during influenza virus infection [11]. Similarly, several works reported the suppression of NRF2 also during SARS‐CoV‐2 infection. In particular, Olagnier et al. [12] demonstrated that the expression of NRF2‐dependent genes is suppressed in biopsies from COVID‐19 patients and the treatment with the NRF2 agonists, 4‐octyl‐itaconate (4‐OI) and dimethyl fumarate (DMF), induces a cellular antiviral program through a Type I IFN‐dependent mechanism that inhibits SARS‐CoV‐2 replication; another study reported that, by using two mouse models of SARS‐CoV‐2 infection, the absence of NRF2 significantly increased viral load and altered inflammatory responses [13]. Our group reported a downmodulation of NRF2 activation mediated by the SARS‐CoV‐2 ORF6 accessory protein [14]; others demonstrated the decrease of the antioxidant gene expression during the infection [15] and a potential role of the nonstructural protein NSP14 in inhibiting the expression of NRF2‐mediated antioxidant genes [16]. Finally, SARS‐CoV‐2 ORF3a and the S protein have been also implicated in the destabilization and degradation of NRF2 [17, 18].

Interestingly, the generation of ROS is also involved in triggering inflammatory responses [19]. Moreover, the NRF2 pathway regulates many other cellular processes, including metabolism and inflammation [20]. A link between NRF2 and antiviral responses has been supposed by its ability to inhibit key signaling components of IFN‐inducing pathways, including ISGs and mitochondrial antiviral signaling [21]. Accordingly, we recently observed a relatively low transcriptional activation of NRF2 and heme oxygenase 1 (HO1) expression in an age‐homogeneous group of hospitalized children affected by respiratory syncytial virus (RSV) severe bronchiolitis [22]. In these patients, there was also higher expression of ISGs compared to the counterpart human rhinovirus (HRV) positive children, strongly supporting the concept that the impairment of the NRF2‐mediated antioxidant pathway leads to an increased inflammatory response [22].

The antioxidant response, in terms of NRF2 protein expression, oxidative stress index, total oxidant status, and total antioxidant status in the serum of pediatric patients affected by SARS‐CoV‐2, was reported by Gumus et al. [23], who found a significant increase in oxidative stress in symptomatic cases compared to asymptomatic children. However, they did not correlate the oxidative stress conditions with the virus‐induced antiviral response.

In the mucosal antiviral innate response, Type III IFNs play a key role due to the specific expression of their receptor in epithelial cells and some immune cells, whereas the Type I IFNs (IFNα and IFNβ) are mainly involved in the control of systemic infection but are also activated in mucosal tissues [24]. The kinetics of the IFN responses are different: Type I IFNs induce many ISGs more rapidly and at higher levels compared to Type III IFNs, which show a delayed and lower induction of ISGs with more limited tissue damage [24]. In children and adults who developed mild coronavirus disease‐19 (COVID‐19), a rapid and effective mucosal innatecoronavirus diseas response has been shown to be critical for control of SARS‐CoV‐2 infection [25, 26, 27]. Indeed, prolonged activation of both Types I and III IFNs may play a detrimental role in lung repair and enhance inflammatory processes in COVID‐19 [28, 29].

The activation of IFN pathways in relation to the antioxidant response in pediatric mild SARS‐CoV‐2 infections has been less studied than in adult infections. Hence, in the present study, we measured the expression of NRF2‐related antioxidant and IFN‐related genes in respiratory samples from subjects attending the pediatric hospital to perform SARS‐CoV‐2 molecular tests. Results were analyzed by comparing SARS‐CoV‐2‐positive and ‐negative samples. Furthermore, the SARS‐CoV‐2‐positive patients were compared according to their symptoms and age to evaluate the antioxidant pathway and to correlate these data with the IFN response.

## 2 Materials and Methods

### 2.1 Participants and Study Design

Children/adolescents presenting for SARS‐CoV‐2 molecular testing at a pediatric outpatient clinic of “Umberto I,” Sapienza University Hospital, Rome, were enrolled after written parental consent in the period from October 2020 to October 2021. Children and adolescents up to 20 years of age were tested because of previous contact with a SARS‐CoV‐2‐positive case within their family or at school, or because of respiratory symptoms. The occurrence of any symptom associated with SARS‐CoV‐2 infection was reported by the parents of the children or self‐reported by the adolescents during follow‐up visits. The study was approved by the Institutional Review Board and the Ethics Committee (Policlinico Umberto I Hospital, Sapienza, University of Rome, Rif. 5836, Prot. 0690/2021).

SARS‐CoV‐2 detection was performed in the University Hospital diagnostic laboratory from nasopharyngeal (NP) swabs as described [30]. Residual NP samples were centrifuged, pelleted with guanidine isothiocyanate reagents, and stored at −80°C; they were included in the study as cases if they tested positive for SARS‐CoV‐2 or as controls if they tested negative.

Subjects who tested positive for SARS‐CoV‐2 were completely asymptomatic or exhibited at least one of the following symptoms: rhinitis, asthenia, lethargy, myalgia, epigastralgia and nausea, and chemosensory disorders (anosmia and ageusia). The presence or absence of symptoms was reported for 26 individuals. None of the SARS‐CoV‐2‐positive children/adolescents had severe COVID‐19 or were hospitalized.

### 2.2 Quantification of mRNAs by qPCR

De‐frozen residual NP cells were subjected to total RNA extraction using RNA‐extraction kits (Norgen Biotek Corporation, Canada); reverse transcription (RT) was performed with 200 ng of purified RNA using the High‐Capacity cDNA Archive Kit (Applied Biosystems, Monza, Italy). Gene expression of NRF2 and redox‐related genes G6PD and APE1 [31] and of the inflammasome NLRP3 (NOD‐, LRR‐, and pyrin domain‐containing protein 3) [32] was determined by qPCR reactions as described in Sorrentino et al. [22]. Briefly, the SensiFAST SYBR NO‐ROX Kit (Meridian Bioscience), was used according to the manufacturer's protocol. The amplification was performed using the iQ5 BIO‐RAD Multicolor Real‐Time PCR Detection System.

The following are primers:

NRF2 forward 5′‐CGTTTGTAGATGACAATGAGG‐3′ and reverse 5′‐AGAAGTTTCAGGTGACTGAG‐3′;

G6PD forward 5′‐CGTCACCAAGAACATTCAC‐3′ and reverse 5′‐GGAGATGTGGTTGGACAG‐3′;

APE1 forward 5′‐CCAGCCCTGTATGAGGACC‐3′ and reverse 5′‐GGAGCTGACCAGTATTGATGAGA‐3′;

NLRP3 forward 5′‐AGGAGGACTTCGTGCAAAGG‐3′ and reverse 5′‐GTGACTCCACCCGATGACAG‐3′ were used.

From the same cDNA, the expression levels of genes coding for IFNα2, IFNβ, and IFNs λ1‐3 and of the well‐known markers of Type I and III IFNs' activation, the ISG15 and ISG56, were measured by quantitative RT‐Real‐time PCR assays [30, 33]. Relative quantification of mRNA levels was calculated with the widely used threshold cycle (*C*_t_) relative quantification method (the $2^{-∆C_{t}}$ method), determining mRNA levels of each target gene relative to the level of the housekeeping gene β‐glucuronidase (GUS) [30].

### 2.3 Statistical Analysis

Statistical analysis was performed by using the Mann–Whitney (MW) test or Spearman's correlation to compare the mRNAs expression between groups. The Jonckheere–Terpstra (JT) test, a rank‐based nonparametric test, was also used for determining significant trends in gene expression levels among three groups formed on the basis of the symptoms experienced during SARS‐CoV‐2 infection (ordered by severity): asymptomatic, symptomatic presenting with mild respiratory or systemic symptoms, and the symptomatic presenting any respiratory or systemic symptoms and anosmia and/or ageusia. Values with *p* ≤ 0.05 were considered statistically significant. The software used was SPSS Statistics v. 27.

## 3 Results

### 3.1 SARS‐CoV‐2 Detection and Clinical Diagnosis in the Study Groups

As reported in Table 1, the study group consisted of 64 subjects under the age of 20 years, including 22 females (34%) and 42 males (66%), with a mean age ± standard deviation (SD) of 10.14 ± 5.43 years. Of the enrolled subjects, 31 were positive for SARS‐CoV‐2 and 33 were negative for SARS‐CoV‐2 and other respiratory viruses.

**Table 1:** Demographic characteristics of study participants.

Items	Total subjects (*N* = 64)	SARS‐CoV‐2 positive (*N* = 31)	SARS‐CoV‐2 negative (*N* = 33)
Sex^a^			
Female, *N* (%)	22/64 (34.4)	13/31 (41.9)	9/33 (27.3)
Male, *N* (%)	42/64 (65.6)	18/31 (58.1)	24/33 (72.7)
Mean age in years^b^	10.14 ± 5.43	9.96 ± 5.41	10.3 ± 5.53
< 12 years, *N* (%)	37/64 (57.8)	18/37 (48.6)	19/37 (51.4)
≥ 12 years, *N* (%)	27/64 (42.1)	13/27 (48.1)	14/27 (51.9)

^a^ Data refer to sex assigned at birth.

^b^ Mean ± SD (standard deviation).

In the SARS‐CoV‐2‐positive group, the mean age ± SD was 9.96 ± 5.41 years; in the negative group, the mean age ± SD was 10.3 ± 5.53 years. In the positive group, 13 were female (42%) and 18 were male (58%). In addition, as described in Methods, clinical data were available for 26 positive patients: 10 (38.5%) were asymptomatic and 16 (61.5%) had mild symptoms.

### 3.2 Comparison of Expression Levels of Antioxidant and IFN‐Related Genes Between SARS‐CoV‐2‐Positive and ‐Negative Subjects

In the NP samples from 64 enrolled subjects, we measured the expression of the following redox genes: NRF2, G6PD, and APE1. The expression levels of the antiviral mediators, Type I IFNs (IFNα2 and IFNβ), Type III IFNs (IFNλ1, IFNλ2, and IFNλ3), the ISGs, ISG15, and ISG56, as well as of inflammasome component NLRP3, were also measured.

**Figure 1:**
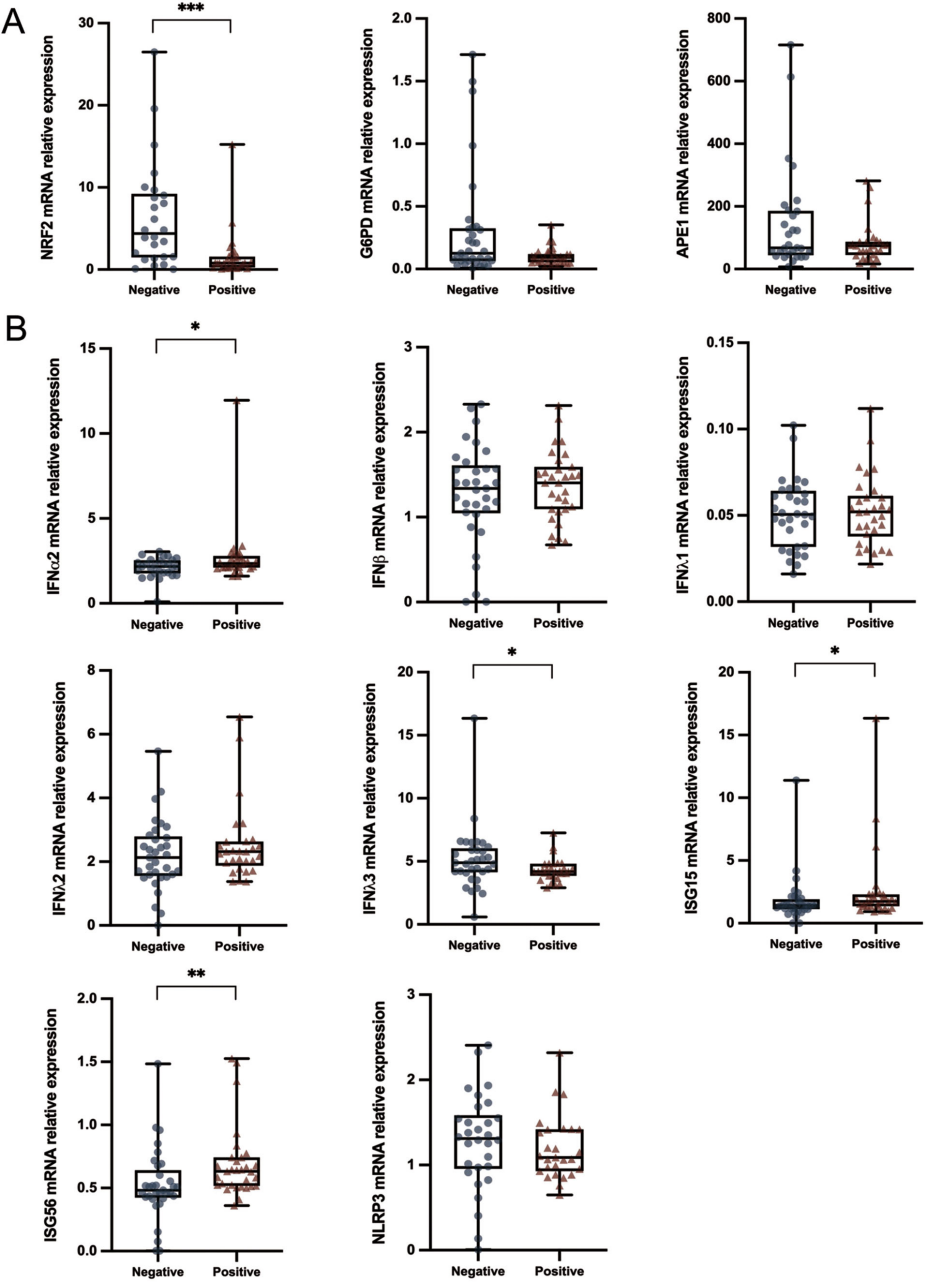
In SARS‐CoV‐2‐positive subjects the expression of NRF2 is down‐modulated, while ISGs are upregulated compared to negative subjects. The mRNA expression values of NRF2, G6PD, and APE1 (A), Type I IFNs (IFNα2 and IFNβ), Type III IFNs (IFNλ1, IFNλ2, and IFNλ3), ISGs (ISG15 and ISG56), and NLRP3 (B), measured by qPCR as described in methods, are depicted as blue spots for SARS‐CoV‐2‐negative and as red spots for SARS‐CoV‐2‐positive samples. Data were compared by the Mann–Whitney *U* test (*p ≤ 0.05; **p < 0.01; ***p < 0.001 vs. negative group).

We first evaluated the antioxidant response measured in samples from SARS‐CoV‐2‐negative or ‐positive subjects. As shown in Figure 1A, NRF2 expression was significantly lower in the positive group with respect to the negative controls. Furthermore, although not statistically significant was also lower in infected patients compared to negative ones, thus indicating an impairment of the NRF2‐related pathway during SARS‐CoV‐2 infection.

Then, the mRNA levels of Type I IFNs, Type III IFNs, ISGs, and NLRP3 were also compared in both groups. A few differences were found between the groups (Figure 1B): mRNA expression of IFNα2 and IFNλ3 was slightly up‐ (MW test, *p* = 0.055) or downregulated (MW test, *p* = 0.038), respectively, in the positive group compared to the negative one. In addition, the expression of ISG15 and ISG56 was upregulated in the infected group (MW test, *p* = 0.049 and *p* = 0.003, respectively, Figure 1B). The NLRP3 gene expression was similar among SARS‐CoV‐2‐positive and ‐negative subjects.

### 3.3 Correlation Between Expression Levels of NRF2 and IFN‐Related Genes Following SARS‐CoV‐2 Infection

**Figure 2:**
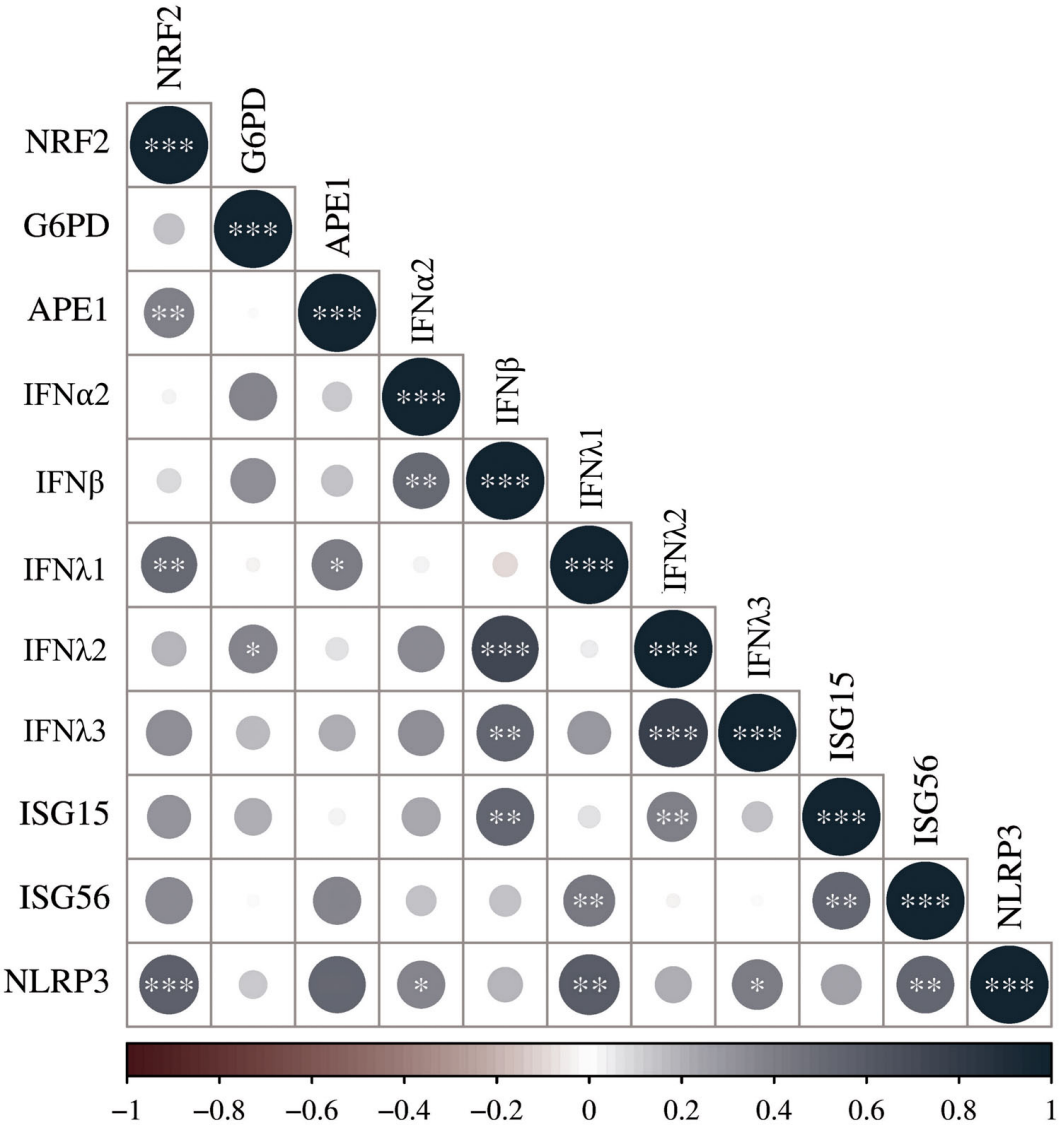
Correlations between NRF2‐mediated antioxidant response and IFN pathways in SARS‐CoV‐2‐positive subjects. Correlation matrix between NRF2, G6PD and APE1, the Types I (IFNα2, IFNβ), and III (IFNλ1, IFNλ2, IFNλ3) IFNs, the ISGs (ISG15, ISG56), and NLRP3 mRNAs in SARS‐CoV‐2‐positive subjects. Spearman's rank correlations are represented by red (negative correlation) and blue (positive correlation) color gradients. *p < 0.05, **p < 0.01, ***p < 0.001.

To determine the biological significance of the induction/repression of the target genes during SARS‐CoV‐2 infection, correlations between their expression levels were evaluated (Figure 2).

First, we found a positive correlation between NRF2 and APE1 (*r* = 0.523, *p* = 0.004). Additionally, a positive correlation was found between NRF2 and the Type III IFNs, IFNλ1, and λ3 (*r* = 0.519, *p* = 0.004 and *r* = 0.364, *p* = 0.053, respectively); moreover, a strong positive correlation was shown between NRF2 and NLRP3 gene levels (*r* = 0.656, *p* < 0.0001).

Furthermore, IFNα2 values were positively correlated only with those of IFNβ (*r* = 0.518, *p* = 0.003) and NLRP3 (*r* = 0.397, *p* = 0.040), whereas IFNβ expression was strongly correlated also with the Type III IFNs (IFNλ2 and λ3; r = 0.829, *p* < 0.0001 and *r* = 0.618, *p* < 0.0001, respectively) and with ISG15 (*r* = 0.536, *p* = 0.002). These findings suggest a common regulatory mechanism governing the expression of these genes in response to infection. Differently, IFNλ1 levels were correlated with ISG56 (*r* = 0.506, *p* = 0.004) and NLRP3 (*r* = 0.559, *p* = 0.003), whereas IFNλ2 and λ3 expression levels showed a strong positive correlation with each other (*r* = 0.724, *p* < 0.0001). Finally, the expression levels of ISG56 and ISG15 showed a reciprocal correlation (*r* = 0.531, *p* = 0.002); the two ISGs were positively associated with NLRP3: ISG56 strongly (*r* = 0.591, *p* = 0.001) and ISG15 weakly (*r* = 0.365, *p* = 0.061).

### 3.4 APE1, Type I IFN Genes, and NLRP3 Are More Expressed in Symptomatic Patients Presenting Anosmia and/or Ageusia

Previous studies have shown that the occurrence of symptoms in children and the need for hospitalization in adults are associated with higher transcription of several IFN genes [30, 34, 35]. However, little is known about their relationship with antioxidant pathway genes, so we sought to analyze our measurements based on symptoms experienced during SARS‐CoV‐2 infection. Hence, the positive patients were stratified into three groups: the asymptomatic (*n* = 10), the symptomatic that had mild respiratory or systemic symptoms including cough, rhinitis, asthenia, lethargy, myalgia, epigastralgia, and nausea (*n* = 8), and the symptomatic that experienced any respiratory or systemic symptoms together with anosmia and/or ageusia (*n* = 8). As mentioned in methods, stratification analysis was performed in 26 patients since data from five positive subjects were not available.

**Figure 3:**
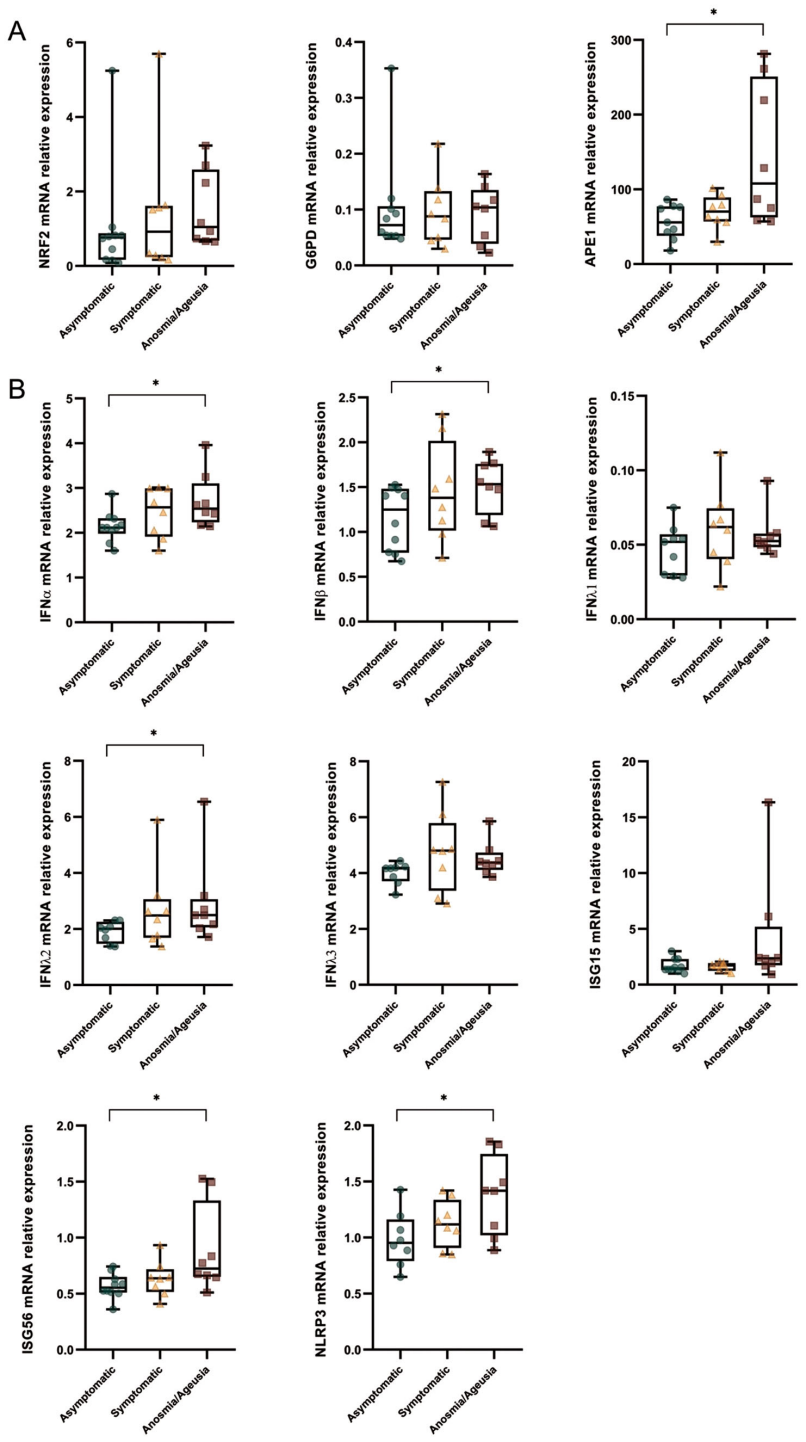
APE1, Type I IFNs, IFNλ2, ISGs, and NLRP3 gene expression is increased in the symptomatic subjects with anosmia/ageusia compared to the asymptomatic group. The mRNA expression of redox genes (A) and Type I IFNs, Type III IFNs, ISGs, and NLRP3 (B) were evaluated by qPCR as described in methods and depicted as green spots for the asymptomatic group, as orange spots for the symptomatic group, and as red spots for symptomatic subjects with anosmia/ageusia. Data were compared by JT test for trends. *p < 0.05.

As shown in Figure 3A, NRF2 and G6PD gene expression values did not significantly differ in the three groups. In contrast, APE1 expression was significantly modulated in the groups (JT test, *p* = 0.032), with the lowest levels detected in the asymptomatic group and the highest levels in the symptomatic group with anosmia and/or ageusia.

Analysis of Type I IFNs showed higher levels in the symptomatic groups (IFNα2, JT test, *p* = 0.030; IFNβ, JT test, *p* = 0.048) (Figure 3B). The same trend was found when comparing IFNλ2 transcript levels (JT test, *p* = 0.042). Values of ISGs, especially ISG56 (JT test, *p* = 0.011), showed progressively higher levels in the symptomatic group and more in the anosmia/ageusia group (Figure 3C). The same increasing trend was found for NLRP3 expression (JT test, *p* = 0.017, Figure 3C).

There was no difference in sex between the three groups, but there was a significant difference in age (JT test, *p* = 0.004). In particular, the symptomatic group with anosmia and/or ageusia had a higher mean age ± SD (14.91 ± 2.1 years) compared to the other group with mild symptoms (8.77 ± 4.5 years; *p* = 0.007) or compared with the asymptomatic group (9.45 ± 3.7 years; *p* = 0.015). It is known that most pediatric patients have mild or asymptomatic illness following SARS‐CoV‐2, and children are much less likely than adults to develop severe COVID‐19 [36]. As the infected group included subjects from 1 to 20 years of age, we sought to analyze gene expression differences after stratification by age. Accordingly, the SARS‐CoV‐2‐positive patients were stratified into two groups, consisting of children < 12 years (*n* = 18, range 0.95–11.90) and adolescents/young adults ≥ 12 years (*n* = 13, range 12.78–19.81). The analysis showed significant differences between the two age groups only in the APE1 gene, which was more expressed in the over 12 years group compared to the children (*p* = 0.003).

Finally, since the patients with anosmia/ageusia had higher mean age, as already described, we wondered whether the expression levels of studied genes were correlated with the age of the enrolled subjects. Age showed a positive correlation only with NRF2 (*r* = 0.382, *p* = 0.034), the redox‐related gene APE1 (*r* = 0.575, *p* = 0.001), and with IFNλ1 (*r* = 0.408, *p* = 0.025).

## 4 Discussion

Oxidative stress plays a pivotal role in the control of respiratory virus infections [11, 12, 37], including those caused by SARS‐CoV‐2. Indeed, a reduction in antioxidant response was observed in biopsies from severely ill patients with COVID‐19, as reported by Olagnier et al. [12]. Additionally, our recent findings demonstrated the role of SARS‐CoV‐2 protein ORF6 in the induction of redox changes, including increased ROS production and impaired NRF2 signaling [14]. Nevertheless, most studies have been conducted in hospitalized adult patients and very little is known about the interplay of the antioxidant pathways with the IFN antiviral response in mild infections.

In the present paper, we evaluated the expression of some antioxidant genes in NP swabs from unvaccinated patients who presented at the pediatric hospital to perform a SARS‐CoV‐2 molecular test. In parallel, the expression of IFN genes and ISGs was evaluated to correlate the modulation of the redox pathway to the inflammatory response in different groups. For this purpose, subjects were first stratified according to positive or negative test results and then the SARS‐CoV‐2‐positive subjects were stratified according to symptoms and age (under and over 12 years).

Although the downregulation of NRF2 at mRNA or protein levels has been previously demonstrated by other groups [12, 23], our findings confirmed that SARS‐CoV‐2‐positive patients with mild disease had a reduction of the antioxidant response in terms of NRF2 mRNA expression. Furthermore, in this study, we found a weak reduction of another redox gene, G6PD, which is involved in the regeneration of NADPH and therefore in regulating the intracellular GSH level [11]. In the positive group, we did not detect relevant differences compared to the control group, in the expression of different IFNs, indicating their moderate transcriptional activation after SARS‐CoV‐2 infection. Consistently, Types I and III IFNs did not show significant activation in respiratory samples from children/adolescents up to 16 years of age, following SARS‐CoV‐2 infection in our previous study [30]. In addition, Gilbert et al. [2] reported that IFNα, IFNβ, and IFNλ2/λ3 expression was unaffected by SARS‐CoV‐2 in pediatric respiratory samples (≤ 15 years), while they found a selective upregulation of IFNλ1 expression at pediatric but not at older ages.

Interestingly, in the present study, ISG56 and, to a lesser extent, ISG15 genes were both upregulated in the positive group. This may be related to the fact that ISGs are also activated independently through the IFN pathway by direct stimulation by Toll‐Like Receptors [38]. In addition, the results of this work are in line with our previous study, where we found activation of ISGs in NP swabs from pediatric patients with severe RSV infection compared with those affected by less severe HRV infection [22].

Stratification of SARS‐CoV‐2‐positive patients into three groups based on the occurrence of specific symptoms showed trends in the expression of APE1, Type I IFNs, IFNλ2, ISGs, and NLRP3, which progressively increased with symptom severity. Indeed, the expression of these genes was lower in the asymptomatic group, increased in the symptomatic patients, and was highly expressed in the symptomatic group with ageusia/anosmia. Differently, Gilbert et al. [2] did not find an association between IFN gene expression and symptoms. The apparent discrepancy in the results might be explained by the different choice in the stratification of the population, including age and type of symptoms. In fact, our patients were younger than 20 years of age and had mild symptoms.

Furthermore, looking at NRF2 antioxidant response, we did not find significant differences in the expression of NRF2 and G6PD among the three groups.

Next, the expression of APE1 was upregulated in symptomatic patients and those with ageusia/anosmia, and its expression positively correlated with NLRP3, although not significantly. APE1/REF1 is a multifunctional enzyme that plays a key role in the base excision repair pathway, and its redox function has been implicated in the activation of several redox‐dependent transcription factors, including activator protein‐1 (AP‐1), nuclear factor kappa B (NF‐κB), hypoxia‐inducible factor 1‐α (HIF1‐α), and signal transducer activator of transcription 3 (STAT3) [39]. In our study, the upregulation of APE1 in positive patients, especially those with more symptoms, is consistent with the findings of Tang et al. [40], who concluded that APE1 regulates NLRP3 expression through the NF‐κB activation. Since APE1 expression was found to be overexpressed in subjects with more symptoms, its inhibition could be suggested as an anti‐inflammatory target to alleviate infection‐related symptoms.

Regarding the APE1/NRF2 correlation, the literature on this topic remains controversial. Some authors found that repression of APE1 led to activation of NRF2 and its function by acting at the transcriptional level, in all cell types tested [41]. In contrast, Shan et al. [42] found that under oxidative stress, APE1 is a key regulator of NRF2‐related antioxidant genes through a redox‐dependent protein–protein interaction in the nucleus. Since we found a positive correlation between the two genes in the positive patients, as reported by the correlation matrix (see Figure 2), our findings deserve further investigation to elucidate the mechanisms underlying their regulation during respiratory virus infection.

Interestingly, although we confirmed NRF2 downregulation in respiratory samples positive to SARS‐CoV‐2 compared to the negative ones, we found a positive correlation between NRF2 and NLRP3 expression in subjects positive to the virus. Both NRF2 and NLRP3 are associated with stress conditions, and several reports have described the crosstalk between these two pathways during the inflammatory process, highlighting the anti‐inflammatory role of NRF2 induction [43]. Therefore, our results suggest that in children, the activation of the inflammatory response to SARS‐CoV‐2 infection, as indicated by the induction of NLRP3 gene expression, is, however, accompanied by a protective induction of NRF2 expression. This is supported by the mild symptoms in the positive group in our clinical data.

In the symptomatic group with anosmia/ageusia, the mean age was higher compared to asymptomatic one, suggesting a relatively more severe infection in adolescents than in young children. Accordingly, Púa Torrejon et al. [44] reported a higher prevalence of anosmia/hypogeusia in adult patients compared to children, although the latter presented these symptoms with a longer duration. Taken together, our results may indicate that SARS‐CoV‐2 infection differentially modulates the expression of antioxidant genes in children of different ages, leading to a differential induction of the host antiviral innate response.

However, our study has several limitations: first, the relatively small number of NP samples and the lack of samples from recovered patients or follow‐up samples obtained after viral clearance. The availability of these samples could have provided insight into the kinetics of antioxidant defense in relation to the IFN response induced by SARS‐CoV‐2. We also recognize that the complex regulatory mechanisms acting on the NRF2 pathway depend on the time since infection and the redox context of the infected cells, which depends on several host immune genes [45, 46, 47]. The expression of immune genes, in turn, is genetically regulated, but also age‐related.

Despite these limitations, we have, for the first time to our knowledge, characterized the mucosal expression of NRF2, three NRF2‐related genes (G6PD, APE1, and NLRP3), IFNs Types I and III, and two ISGs, known markers of IFN activation, in the context of mild SARS‐CoV‐2 pediatric infections from diagnostic specimens.

In conclusion, our results are consistent with the notion that SARS‐CoV‐2 infection inhibits NRF2 expression in the upper respiratory tract in pediatric subjects. Moreover, the data showed that in asymptomatic subjects, NRF2 is less expressed compared to groups with symptoms, in which inflammatory genes are upregulated. This is consistent with the previous finding in which the occurrence of symptoms in children was associated with higher transcription of several IFN genes [30] and supports the close link between inflammatory responses and the antioxidant pathway. Indeed, NRF2 is a key regulator of the antioxidant response of the cell, and it has been shown to widely control the innate immune response. Several respiratory virus infections, commonly associated with increased oxidative stress, affect the NRF2 pathway and consequently the host antiviral response. This study highlights the role of NRF2 and related genes in regulating the IFN response in SARS‐CoV‐2‐positive children. The observed suppression of NRF2 and its correlation with high increases of IFNα2 and IFNλ3 response, along with increased expression of ISGs, provide valuable insights into the host response to the virus in the young population. Further investigations are warranted to explore the potential of NRF2 modulators as therapeutic strategies for COVID‐19, particularly in children.

## Author Contributions

**Toscanelli Walter:** conceptualization, methodology, formal analysis and investigation, data curation, writing–original draft preparation. **Fracella Matteo:** methodology, formal analysis and investigation, software, data curation, writing–original draft preparation. **De Angelis Marta:** conceptualization, formal analysis and investigation, data curation, writing–original draft preparation, writing–review and editing. **Scagnolari Carolina:** conceptualization, supervision. **Sorrentino Leonardo:** methodology, investigation, formal analysis. **Piselli Elena:** methodology, investigation. **Marcocci Maria Elena:** methodology, investigation. **Midulla Fabio:** supervision, funding acquisition. **Mancino Enrica:** methodology, investigation. **Nenna Raffaella:** methodology, validation, formal analysis. **Petrarca Laura:** methodology, validation, formal analysis. **Palamara Anna Teresa:** supervision, funding acquisition and project administration. **Antonelli Guido:** supervision, funding acquisition and project administration. **Pierangeli Alessandra:** conceptualization, writing–review and editing, supervision; **Nencioni Lucia:** conceptualization, writing–review and editing, supervision, funding acquisition and project administration. All authors have read and agreed to the published version of the manuscript.

## Conflicts of Interest

The authors declare no conflicts of interest.

## Data Availability

The authors have nothing to report.
